# Does Colour Filling-In Account for Colour Perception in Natural Images?

**DOI:** 10.1177/2041669518768829

**Published:** 2018-05-07

**Authors:** Christopher W. Tyler, Joshua A. Solomon

**Affiliations:** City University of London, London, UK

**Keywords:** colour, contours/surfaces, filling-in, perceptual organization

## Abstract

It is popular to attribute the appearance of extended colour fields to a process of filling-in from the differential colour signals at colour edges, where one colour transitions to another. We ask whether such a process can account for the appearance of extended colour fields in natural images. Some form of colour filling-in must underlie the equiluminant colour Craik–O’Brien–Cornsweet effect and the Watercolour Effect, but these effects are too weak to account for the appearance of extended colour fields in natural images. Moreover, the graded colour disappearance effect reported as evidence for colour filling-in does not work under natural viewing conditions. We demonstrate that natural images do not look very colourful when their colour is restricted to edge transitions. Moreover, purely chromatic images with maximally graded (edgeless) transitions look fully colourful. Consequently, we conclude that colour filling-in makes no more than a minor contribution to the appearance of extended colour regions in natural images.

There is fairly widespread acceptance of the notion that extended fields appear colourful more from colour filling-in from the colour changes at the edges of the field than from the inherent colour of the field itself (Gerrits & Vendrik, 1970; Kanai, Wu, Verstraten, & Shimojo, 2006; Larimer & Piantanida, 1988; Paramei & Van Leeuwen, 2016; Pessoa, Thompson, & Noë, 1998). At least this idea is the basis for many computational analyses of colour processing (e.g. Grossberg & Mingolla, 1985; Ma & Manjunath, 2000). The argument proceeds from the proposal by Craik (1966), O’Brien (1958) and Cornsweet (1970) that lateral inhibition reduces or eliminates the neural response to uniform fields, leaving as the most salient features the edges, or regions of rapid luminance change in the image. (The lateral inhibition is considered to be implemented neurally by surround inhibition balancing the centre activation of typical neural receptive fields.) The luminance of the uniform regions is then supposed to be regenerated by the somewhat mysterious process known as *filling-in*, by which the peak luminance on each side of the luminance edge is proposed to propagate throughout the uniform field regions between the edges. This concept is then generalized from luminance to colour fields, as quantified by Wachtler and Wehrhahn (1997), who showed that it averaged about 10% of the strength of the luminance filling-in, or about a 0.5% effect (in terms of cone contrast). To be fair, the maximum range of chromatic modulation in terms of cone contrast is only about 15% (Cavanagh, Tyler, & Favreau, 1984); so a 0.5% luminance effect could translate to about 3.5% of the full range of chromatic modulation, but this still leaves 96.5% of the perceived chromaticity of extended 100% colour fields to be accounted for.

Along similar lines, colour filling-in is the conceptual basis of the ‘Watercolour Effect’ of Pinna (1987), in which colour is indeed seen to propagate across the white spaces between the double lines of two contrasting colours (Figure 1, left panel). (Interestingly, the strength of the effect is dependent on both the line wiggliness and the luminance difference between the colours, as illustrated by the straight-line, equiluminant version in Figure 1, right panel.) Pinna, Brelstaff, and Spillmann (2001) report that its strength increases with the spatial frequency of the wiggliness of the coloured borders, although perfectly straight borders also induced a notable effect, as seen in Figure 1 in a figure/ground configuration. However, they do not offer any explanation for the wiggliness effect. Pinna, Werner, and Spillmann (2003) describe the role of a range of Gestalt factors in the effect, but do not include straight-line versions in their analysis. Thus, it is undeniable that there is a colour filling-in mechanism operating in human perception, and as such is deserving of a full analysis of what neural mechanisms could account for the immediate propagation of edge-defined colour across extensive spatial regions (Komatsu, 2006). However, though spatially extensive, the colour effect is markedly unsaturated, so we raise the question whether it can account for the normal range of colour perception of extended surfaces or is a minor tweak on the full scope of colour processing.

**Figure 1.**
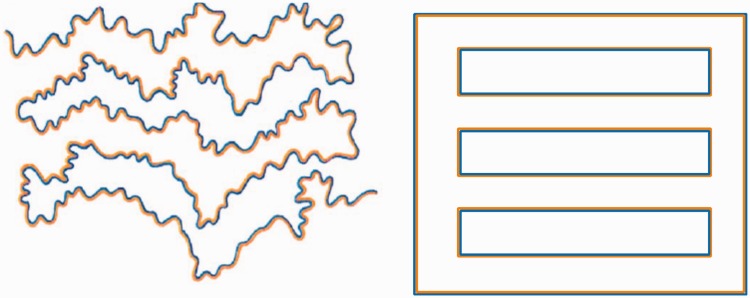
Left panel: An example of the Watercolour Effect of Pinna (1987). Right panel: A straight-line, fully enclosed version with the colours equated for luminance, showing a somewhat weaker effect.

A primary argument against the logic of colour filling-in as a general phenomenon is that many colour-selective receptive fields typically do not exhibit surround inhibition (for review, see Komatsu, 2006). Moreover, there is little or no evidence for lateral inhibition in the colour domain. Colour modulation does not elicit the type of low frequency fall-off in either the spatial (Van Der Horst & Bouman, 1967; van der Horst, de Weert, & Bouman, 1967) or temporal (Regan & Tyler, 1971; van der Horst, 1969) domains that is taken as psychophysical evidence for lateral inhibition in the luminance domain (Campbell & Robson, 1968; De Lange, 1958). These lines of evidence give every reason to expect only a minimal filling-in process to operate in the colour domain. We ask whether, even if colour filling-in operates under laboratory conditions, it is an effective operating mode for the viewing of natural images. It seems most natural to answer this question under natural viewing conditions of free eye movements.

This argument against colour filling-in was, however, challenged by a demonstration by Krauskopf (1963) and others that an ‘edgeless’ blurred red disk within an equiluminant green disk could disappear under stabilized image conditions, with the green appearing to invade the red region to fill it in as a uniform green disk. Examples of such a stimulus are shown in Figure 2 at two scales, both in the original colours and in a blue–yellow version (on the grounds that the latter should perhaps be more vulnerable to colour filling-in because the S-cones coding the blue–yellow colour contrast are about a factor of 10 less dense than the other cones in the retina). It is clear from the images in Figure 2 that, under natural observation conditions with free eye movements, there is no significant reduction in the colour of the blurred centre relative to the sharp-edged surround, even with the steadiest fixation effort. Under natural viewing conditions, then, there seems to be no substantial colour filling-in for the Krauskopf-type ‘edgeless’ colour stimulus. This too was noted by Krauskopf. It may be possible to approximate Krauskopf’s mechanical stabilisation with exceptionally stable fixation for 20 s or more, but this is a manifestation of general adaptation at low spatial frequencies, reducing the already limited contrast sensitivity below the colour discrimination threshold. It is therefore clear that the green and blue edges do not propagate their colours into the centres of the fields under conditions of normal saccadic exploration.

**Figure 2.**
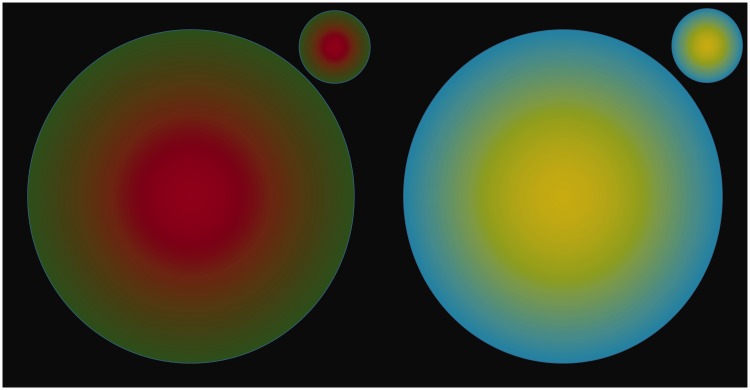
Krauskopf style ‘edgeless' red and yellow colour disks in roughly equiluminant green and blue surrounds, respectively, at two size scales. Steady fixation fails to eliminate the colour gradient under normal viewing conditions. Note: With prolonged steady fixation and transfer to a blank test field, the complements of the inner colours are again fully visible in the afterimages, despite the fact that afterimages are by definition stabilized on the retina. This implies that the Krauskopf filling-in is a version of the Watercolor Effect at the low effective contrast produced by stabilization, which is ineffective for the high effective contrast afterimages.

The images in Figure 2 are artificial images, but the main question of this article is whether colour filling-in might operate in natural images under more natural viewing conditions, given the complexity of their edge structures, mixture of chromatic and luminance edges and contextual richness. For a direct assessment of colour filling-in in natural images, we chose a colourful macaw image. The base equiluminant stimulus was generated by the procedure shown in Figure 3. In the first column are the original image (upper panel), with the greyscale information extracted by setting the colour contrast to zero (lower panel). Subtracting the greyscale image contrast in each colour channel from the original produced the equiluminant colour image of the second column (upper panel). Setting the colour contrast of this equiluminant image to zero without changing its luminance contrast (second column, lower panel) verified that there was minimal residual luminance contrast.

**Figure 3.**
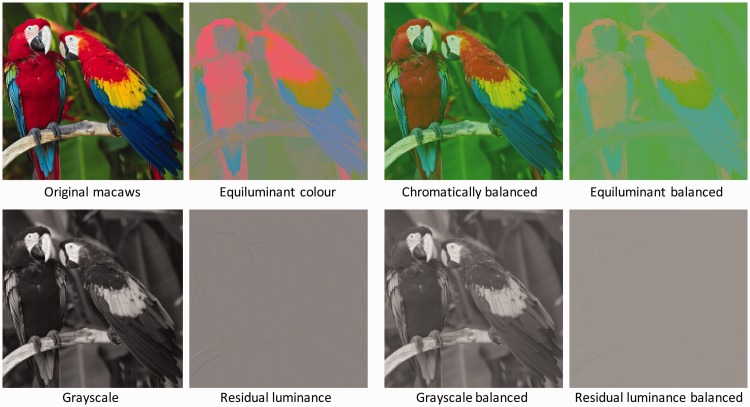
Generation of an equiluminant, balanced contrast colour image (see text). Figure Credit: dima266f.

Now, although this luminance subtraction procedure generated an equiluminant image, it is evident that the colour contrast is not equal everywhere – in particular, the reds and blues in the feathers are much more saturated than the greens and yellows in the background. In an attempt to equate the colours across space, we therefore raised the intensity of the green channel to the point where it provided a perceptual match to the brightness of the other colour channels (third column, upper panel) and repeated the same sequence of operations from the first quartet in the remaining three panels. It can be seen that the resulting equiluminant, balanced image now has much more uniform colour contrast throughout, and that its residual luminance image still has minimal contrast energy. While we did not attempt quantification of these matches, we relied on visual assessment to come close to a perceptual match, and to verify that the residual luminance contrast energy is essentially undetectable.

To evaluate the question of colour filling-in, therefore, we need a high-pass spatial frequency version of the equiluminant, balanced contrast colour image of Figure 3, as provided in the extremely high-pass image of Figure 4 (left panel) filtering the 512x512-pixel image with a 2D Gaussian filter that dropped to 1/e at 7.5 pixels. It can be seen that the image appears as narrow lines of high contrast colour with predominantly uniform grey between them (although there may be a hint of perceived colour as expected from the Watercolour Effect). It is evident, therefore, that the colour borders per se are not sufficient to provide for the full-colour impression of the original image.

**Figure 4.**
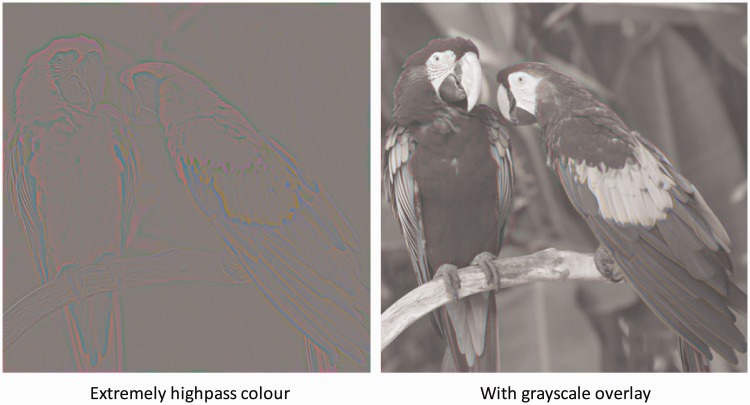
Left panel: High-pass spatial frequency version of the equiluminant, balanced contrast colour image of Figure 3 (fourth column, upper panel). Right panel: Left panel image added to the balanced greyscale overlay of Figure 3 (third column, lower panel). Figure Credit: dima266f.

Now, it could be argued that full-colour equiluminant images are not very natural, even when derived from a natural image. It seemed prudent, therefore, to re-introduce the achromatic information back into the image to see if there was some interaction between it and the high-pass colour information that could provide for some colour filling-in. The effect of this manipulation is shown in Figure 4 (right panel). It is clear that the effect of the luminance information is, if anything, to mask the colour information rather than to induce filling-in, as the colour appears more vivid in the high-pass equiluminant colour image than in the presence of the luminance information.

In addition to subtracting the full achromatic image from the chromatically balanced image (Figure 4), we also subtracted progressively more blurred versions of the chromatic image from itself, leaving progressively more of the mid-frequency chromatic information from Column 1 to Column 3 of Figure 5. With an image dimension of 512 × 512, the equiluminant, balanced colour image was filtered with a 2D Gaussian filter that dropped to 1/*e* at 15, 30, 60 pixels to provide the very, moderately and mildly high-pass filtering, respectively. It can be seen that the image of the first column still looks like narrow lines of high contrast colour with predominantly uniform grey between them (although there is some increase of colour as expected from the Watercolour Effect). In the last two columns, there is a noticeable spread of the colour towards the centre of the smaller regions, but they do not look anything like the full-frequency equiluminant colour image of Figure 3 (fourth column, upper panel), and it is difficult to distinguish any perceptual spread from the actual image spread expected from a more extended band of spatial frequencies as the pass band is lowered. If there is some degree of colour filling-in, it is not the process responsible for the full-colour perception of such an image.

**Figure 5.**
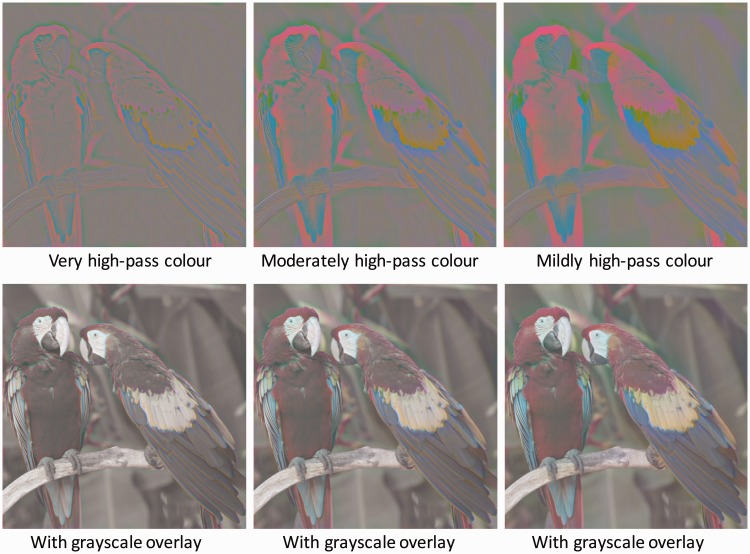
Upper panels: High-pass spatial frequency versions of the equiluminant, balanced contrast colour image of Figure 3 (fourth column, upper panel) at three further high-pass cutoffs. Lower panels: The images of the upper four panels added to the balanced greyscale overlay of Figure 3 (third column, lower panel). Figure Credit: dima266f.

In the lower panels of Figure 5, adding the high-pass equiluminant versions to the greyscale image does seem to make the colour look noticeably more uniform in the presence of the luminance information (lower panels) than in the equiluminant colour images (upper panels). This leads us to the suggestion that colour filling-in can be slightly enhanced by the natural forms of luminance contrast in natural images, although whether this is a true filling-in process or an effective reduction in colour contrast by luminance masking/gain control mechanisms remains unclear.

One objection to this analysis might be that the equiluminant balancing procedure departs from the unbalanced nature of the colour across natural images in general, and that colour filling-in might be more effective for unbalanced colour edges. To address this point, we include a further figure of the colour edge manipulation for the unbalanced equiluminant case from Figure 3 and four other diverse natural images (Figure 6). It is evident that only the smallest colour patches at the scale of the filter show pronounced colour percepts. For more extended regions, any filling-in is markedly incomplete relative to the full-colour versions, providing further support for the contention that colour-edge filling-in is not the primary mechanism for our colour perception in natural images.

**Figure 6.**
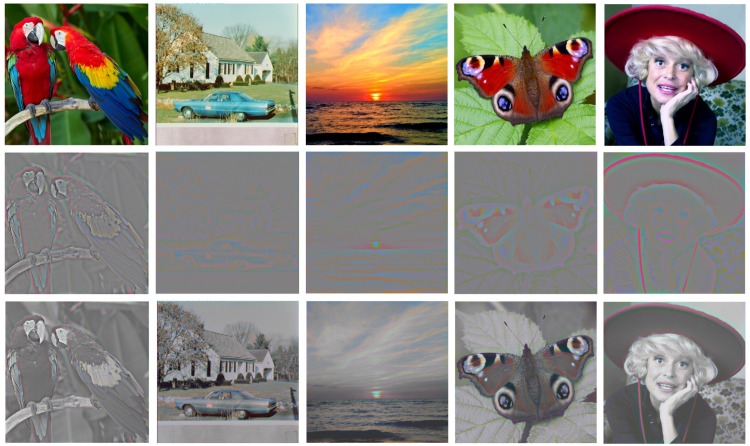
The chromatic edge information for a diversity of natural images (top row) shown as the raw (non-balanced) colour edges at the ‘very high-pass’ level of 1/*e* = 15 pixels in the 512 × 512 images (middle row), and added to the unfiltered luminance information (bottom row). Although full colour is seen for features at the scale of the high-pass filter (as in the turquoise car and Carol Channing’s red hat lanyard), there is rather little percept of filling-in across larger coloured regions in these natural images. Figure Credits (left to right): dima266f, pixelio.de, Fedor Yakubovich, Tony Hisgett, Allan Warren.

## The Role of Spatial Frequency

**Figure 7.**
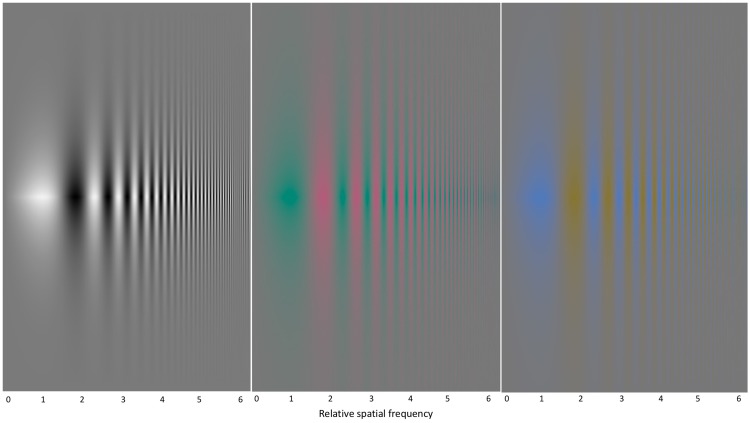
Bivariate swept spatial frequency and contrast gratings on the three axes of the colour space (light/dark, red/green and yellow/blue), configured to eliminate any high-contrast edges. Source: Modified from http://blog.kasson.com/the-last-word/chromaticity-csfs/, downloaded August 1, 2017. Figure Credit: Jim Kasson.

A final issue is the role of spatial frequency in colour processing. It is generally understood that colour processing, as represented by the visibility of equiluminant colour images, is limited to much lower spatial frequencies than luminance processing (National Television System Committee, 1953; Shevell, 2003; Wandell, 1999). However, as can be discerned from the frequency and contrast-modulated gratings in Figure 7, in which maximum contrast appears along the horizontal centre line, the resolution limit for black and white is only about twice as high as that for red/green modulation (van der Horst, de Weert, & Bouman, 1967; Anderson, Mullen, & Hess, 1991). Results for yellow/blue modulation are more variable (Ahumada, Wuerger, & Watson, 2002), but appear to be similar to that for red/green in this configuration. (Care must be taken to ensure that the coloured images are viewed in clear focus, as the focal plane may be different for the optimal viewing of each colour.) This novel configuration is designed to eliminate any sharp edge information, either luminant or chromatic, from which the interior grating could be reconstructed by a filling-in process. Thus, any colour percept of deviation from the average gray has to derive from the local interior information within each bar of the swept gratings, rather than by propagation from edge information.

The second observation to be drawn from Figure 7 is that the equiluminant sinusoidal colour modulation is equally visible at low spatial frequencies in the absence of any sharp colour or colour/luminance edges from which to propagate a colour signal, that is in the absence of the possibility of colour filling-in. This observation implies that the colour is perceived *sui generis*, solely on the basis of the available colour gradients in the sinusoidal modulations, as exemplified by Figure 7. It is not clear how colour filling-in proponents would account for the colour impressions of such sinusoidal colour modulation, but our view is that it leaves at best a minimal role for any such nonlinear chromatic processing.
